# The Nature and Treatment of the Opium Habit

**Published:** 1889-09

**Authors:** Homer E. Smith

**Affiliations:** Atlanta, Ga.


### THE NATURE AND TREATMENT OF THE OPIUM HABIT.

Read before the Atlanta Society of Medicine.

BY HOMER E. SMITH, M. D., ATLANTA, GA.

The meagerness of detail usually given in works on medicine to opium addiction, and the total inadequacy of its usual treatment, will be my apology for presenting this subject to-night; and also because any method of treatment which can promise to the patient a certainty of cure, and that a painless one, is worthy of your consideration.

However interesting it may be, we cannot enter into the history of opium addiction. Men in all ages of the world have resorted to stimulants and narcotics, and the evil effects of the former have been paraded at length in speech and story, from press and pulpit, until every phase of alcoholic intoxication has been dilated on for a warning against it; while against the more debasing "habit," against the more demoralizing form of intoxication, little has been said, for the opium habit renders its victims less conspicuous to the eyes of the laity, and, being practiced in secret, lays itself less open to censure; but none the less surely does it invade and undermine the body's health, blunt the moral sense and wreck the intellect. The want of proper appreciation of this disease and study of its treatment is due mainly to two factors—one is the secrecy which patients keep in regard to it, from their fear of detection, and the other the idea which is held as to its nature.

The name itself, the opium "habit," implies a vicious intent, a moral obliquity on the part of its victim, instead of a physical infirmity; and feeling this, the *habitué* too often seeks relief from charlatans who thrive on his distress, when, with a right understanding of its true nature and its proper recognition as a toxic neurosis, the patient would more naturally turn to the physician, and he, qualified for an intelligent treatment of it, would be able to materially lessen this great and growing evil. Two factors stand in genetic relation to opium addiction—necessity and desire. As regards my own observation of this disease, I have found that the vast majority date their use of some form of opium from some physical disorder requiring its use (for a period more or less prolonged), which, when its proper mission for the relief of the primal disturbance was ended, had created a demand for continued taking that *would not* be denied. Heredity plays a minor *rôle* as a causal factor, but still one whose influence as a predisposing cause is not to be denied, and which must be taken into account in the prognosis of a relapse after cure. The inherited tendency to inebriety, whether to alcoholic or narcotic excess, is well recognized, and cases are on record of congenital opium addiction. The maternal influence in both cases is the stronger, and infants of opium-eating mothers manifest, on weaning, all the symptoms which follow a discontinuance of the drug in adults accustomed to its use, which will result in death to the little sufferers unless opium be administered to them.

Neuralgia, and other diseases in which pain is a prominent symptom, are frequent sources of opium addiction, and physicians themselves fall frequent victims to the disease. As one writer forcibly attests, "A physician who is afflicted with any of the neuralgias, and who owns a hypodermic syringe, is sure, sooner or later, to become a confirmed taker if he tampers with this seductive and fascinating alleviative." Truly a great responsibility, little realized, rests on the physician who indiscriminately prescribes morphia for every pain or ache, sometimes going so far as even to order a syringe for the patient himself to use as circumstances require. There seems to be an avenging Nemesis in that so many physicians themselves fall under its influence. Occasionally we find cases of opium addiction supervening on alcoholic excess—those given to stimulants resorting to its use in the vain hope of thereby mastering the appetite for alcohol. Rarely does this succeed, but, on the contrary, we find the use of opium engrafted upon that of alcohol with the result of an excessive use of both. Indeed it is truly astonishing to note the amount of whiskey that can be consumed by the chronic opium eater without its manifesting its characteristic effects. I recall the case of a man under my care who, in his normal condition, was peculiarly susceptible to the influence of alcohol, but who could take a quart of whiskey in the course of the morning without any apparent effect. A few cases of opium addiction take their origin in some psychic disturbance, such as grief, anxiety and the mental strain that accompanies the high-pressure business methods in our monetary centers. A New York druggist told me that many of his regular customers were Wall street brokers, and the persistent insomnia that rounds up the day of many of these busy men tempts them to woo Morpheus with morphia. There are other lesser causes for the use of this drug, for instance experimental self-medication, force of example, its use as a stimulant, etc. But these are minor considerations, and there remain more important points to take up. What I would insist on is that—whatever the cause may be—continued taking in a short time places the patient beyond hope of a voluntary relinquishment of its use, converts what *may* have been an indulgence into a necessity, and makes of him a sick man for whose cure intelligent and scientific treatment is needed.

With one exception, there are minor differences in the effects of the drug, no matter how it is to be taken into the system. The exception I refer to is smoking. From this method of use, the disturbances of the nervous system are much more marked, the emaciation more rapid, and the gastric disorders greater. Why this is so is not clearly known, but it is probably due to the fact that morphia and its salts are less volatile than the other opium alkaloids, whose sedative effects are less than their convulsive. Therefore reaction comes on sooner, and a greater quantity is taken in this form than would be necessary of the more narcotic preparations. This form of addiction, however, comes but rarely for treatment. It is the least common method of use, being chiefly confined to the Asiatics and in this country to prostitutes and men of the lowest class. Ranking them in the order of least injuriousness, they stand with the above exception as follows: Hypodermically, *per rectum* and *per os*. In the two latter methods the crude drug creates greater disturbances than the salts of morphia. The symptoms that follow habitual use of opium are prominently manifested in the nervous system. These are due to the sedative effect on the spinal cord, a want of proper reflex action in this organ, and to cerebral disturbances, induced *primarily* by a retention in the blood of effete products of tissue metamorphosis, whereby the brain is denied a healthy pabulum; and *secondarily* by the mal-nutrition in which this organ participates in common with others in connection with the general cachexia. The profound sleep that usually follows the administration of a full dose of opium in one unaccustomed to its use is often, in the *habitué*, accompanied by painful dreams, convulsive twitchings of the extremities, and, later in the disease, by broken rest, and not infrequently insomnia, for which the drug may have been primarily taken, results. If the poison of gout or enteric fever circulating in the system affects the brain, is it surprising that effete animal products retained in the body of the opium eater should cause aberrations of the cerebral functions? Illusions, delusions and hallucinations are not uncommon symptoms. Both the intellect and moral sense are perverted. The brain refuses to do its work under these abnormal conditions, and cerebral processes go on with difficulty. The memory becomes impaired, and reason, without that purveyor, lacks cogency and force. A disinclination to physical and mental efforts ensues, the temper becomes irritable, and indeed I have seriously questioned whether the confirmed taker is of sound mind. Upon the physical economy its effects are not less marked. Profound alvine torpor takes the place of normal intestinal action, the bowels moving only by the use of enemata or strong cathartics. Occasionally intercurrent attacks of diarrhœa set in. This form, called opium diarrhœa, rarely lasts more than a few days, but resists obstinately all the ordinary astringents. It is due to decomposing masses of food, imperfectly digested, lying in the intestinal tract, and is usually accompanied by marked gastric disturbances. As this diarrhœa differs entirely from that which follows the withdrawal of opium and calls for different treatment, I incidentally mention here that it may be controlled by the administration of large doses of castor oil, followed by pepsin and the salicylate of soda. The former removes offending substances; the latter mixture aids digestion and checks fermentative action. The skin of those addicted to opium assumes an earthy hue, the face a deathly pallor. The pupils are contracted. Emaciation rapidly supervenes, unless alcohol be also taken in excess, and then the body assumes a bloated and puffy appearance. The sexual appetite is blunted or lost. The pulse and respiration become slower and weaker. The appetite fails and digestion goes on with difficulty. The gastric juice is secreted in lesser quantity, and functional digestive disorders result. Nausea is a common symptom, and frequently vomiting takes place, either on rising or soon after each meal. The kidneys act slowly and the urine, which is increased by a single dose of opium, becomes scanty and high-colored under its continued use, and albuminuria may sometimes ensue. Every secretion is diminished or almost suppressed except one, that of the the skin,—the coldest day does not check it, and summer heat adds not largely to it. The confirmed taker wakes from a troubled dream to find his body dripping wet, his form outlined in moisture on the sheet, and his pillow damp beneath his head. He must rise for change of clothing, cold chills attack him, he hastens back, loathing himself and the life he is compelled to lead, and morning finds him bathed in sweat again. Nevertheless in this, the most distressing condition to the patient, lies his salvation, for nature, striving to accommodate herself to the abuse of her other eliminations, puts a double increase of duty on this one, for notwithstanding the large increase in the quantity of the perspiration, it will be found to contain more than its usual proportion of the *débris* of tissue metamorphosis. When in the last stages of this disease this elimination also fails, also then will supervene the more serious results which follow the retention of effete products in the animal economy, and coma, convulsions and death will ensue. It is folly to speak of this condition (the picture of which is not overdrawn) as a habit. The chief responsibility indeed with the *habitué* lies in his initiation into, rather than in his continuance of, the use of opium. Leaving aside all considerations of the evil nature, the sin, etc., which well-intentioned but illy-informed moralists prate about, the responsibility now rests with us, as physicians, to hold out to these unfortunates some method of relief that shall induce them to trust themselves to our care, with a confidence born of a knowledge that they will be rationally and scientifically treated for the disease from which they are suffering.

What is the reason that the papers are filled with advertisements of so-called opium cures that promise "a painless cure in ten days," "opium habit cured at home without suffering," etc., etc. It is because the profession has had nothing or next to nothing to offer instead, and even that at an amount of suffering which these emaciated, broken-down and nervous invalids could not or would not endure.

These vampires who thrive on the distress of others, I may dismiss in a very few words.—They are charlatans and cheats to a man, for if there be any drug that is a substitute for and will admit of the total and instantaneous cessation of morphia without pain, I do not know of it. The whole range of the Pharmacopœia has been searched, and now and then we read in the medical journals that the long sought for antidotal drug has been found, but after a more or less prolonged vaunting of its virtues, it proves to be an utter failure and sinks into innocuous desuetude.

Gentleman, have any of you ever stood by the bedside of a confirmed opium-eater, struggling unaided to escape from the pitiless mastery of the sorcery of opium? Let us see what will follow the cessation of the drug in one long accustomed to its use. We will say the last dose has been taken at bedtime and the night is past. The morning comes and with it the first thought is for the accustomed dose. No unpleasant feelings, however, begin to manifest themselves so soon, but during the day a lack of something necessary for his comfort is borne in upon the patient. The lachrymal secretion long-supressed begins again to flow, his eyes are suffused, the tears overflow and, mingling with the nasal secretion, provoke constant blowing of the nose and sneezing. Other reflex symptoms manifest themselves. The spinal cord asserts itself, and contractions and stretching of the limbs alternate with wearying frequency. The feeling of *malaise* is intensified. Appetite is entirely gone and a fatigue more marked than that which follows intense exertion sets in. A restlessness that will not allow a minute's quiet takes possession of him, and no position brings relief. And now night has come again; dispirited, anxious and depressed, he dreads the thoughts of what may follow. He falls asleep, but frightful dreams awake him. Neuralgic pains seek out the site of undiscovered nerves. Hot and cold flashes alternately pass over his body which is dripping at every pore with sweat. The morning *malaise* has deepened into an excruciating distress,—not pain for this alone were easier to bear,—but a peculiar, indescribable anguish torments him from head to foot. Superadded to all this, obstinate vomiting sets in, followed by profuse, exhaustive diarrhœa, and without aid delirium soon ensues. In this condition, if it be from voluntary relinquishment and opium is within reach, how many, knowing that one single dose will lift them from this utter misery into perfect ease and comfort, will abstain? Put yourself in his place and ask yourself what you would do. I lately came across in my reading a pathetic story, which I give entire: A young man, an undergraduate in an eastern business college, had become the victim of the hypodermic use of morphia. He went with his father, who was engaged in the lumbering interest, into the primeval forests of Maine, hoping that during a stay of months with the wood-choppers he would be able to fight out the battle of gradual abandonment successfully. Through a strange fatality when the party had just arrived at their camping place and were transporting their goods across a stream, the case of morphia was broken by an apparent accident and its contents scattered into the water. None but the haggard young man comprehended the appalling magnitude of the calamity. There he was two hundred miles from the nearest settlement. He survived the terrible ordeal, but no words, he has said, could express the tortures and agonies through which he passed during the succeeding weeks. He was closely watched, else at times he would have drowned himself or beaten his brains out on the rocks. Months afterwards he came back to the world, a skeleton, worn and haggard from his terrible contest. It was an experience he could never afterward refer to without the most painful emotions. Not the least significant point in this veritable account is the fact that the young man always believed that his father purposely brought about the catastrophe for the sake of bringing matters to a speedy end.

Has the usual treatment by physicians at this day anything to offer that is much better than this man's summary method? Perhaps no work on this subject has appeared in recent years more thorough in its scientific intention than Dr. Levinstein's "Morbid Craving for Morphia." It is evident that he has brought no common accuracy of observation to bear upon the subject, and his clinical notes are full of interest for the opium *habitué*. There is a striking parallel between the method of the Maine lumberman and advanced German science in the treatment of this disease. In both cases the patient suffers from the intense cruelty of ignorance. The best thing to do for the unfortunate victim of morphia, according to this learned work, is to secure him in rooms under charge of a competent nurse or keeper, his person and baggage having been searched, and from the rooms (using Levinstein's own words) all opportunities for attempting suicide having been removed. Doors and windows must not move on hinges but on pivots; must have neither handles, bolts nor keys, being so constructed that patients can neither open nor shut them, and hooks for looking-glasses, clothes and curtains must be removed. Certainly these are ominous preparations for a course of scientific medical treatment. Within this prison, the patient, guarded by trained nurses who never leave him, is at once deprived of opium in every form and here he must struggle through the terrible weeks succeeding as best he may. So far as it appears, he has but the slightest medical aid. His symptoms, however, are closely watched, night and day, for the portentous shadow of one special danger looms ever near his bedside, that of sudden collapse of the vital powers. A few minutes delay in such a contingency may prevent all powers of resuscitation. In any case the situation is very critical, and well will it be if morphia, which is always the immediate resort in such emergeneies, have not lost its potency. The clinical notes of Levinstein, in form cold and terse as a hardware catalogue, are fairly burning with their burden of tragedy, recounting as they do the story of the tortures through which the patient passes; the days and nights of writhing, the sleeplessness, the thirst, the endless vomitings and purgings, and the vain pleading for liberty, for morphia, for *anything* that will relieve the intolerable anguish.

We now come face to face with two practical questions: If opium addiction curable, and can we do anything to relieve the sufferings of the patient ? To both these questions the answer is emphatically, *yes*, and the keystone of the arch in its successful treatment is preliminary sedation. To this use of sedatives, originality, I think, must be given to Dr. J. B. Mattison, of Brooklyn. The minor details of treatment will be utterly worthless without it, and it is placing reliance on symptomatic medication that has caused so many failures and induced so many physicians, even those who make a specialty of this disease, to give up in despair and to fall back on expectancy, with what results I have shown you in quoting Dr. Levinstein. To make my meaning clearer, hypnotics fail in relieving the sleeplessness; astringents have no power over the diarrhœa; vomiting goes on unchecked in spite of antiemetics; and restlessness does not yield to sedatives, *unless* the chief causal factor in all these conditions—the spinal cord—be primarily controlled. This effected, the rest is easy. To take up the treatment in detail, we need first a trained nurse, one who can intelligently note symptoms, take temperature and count pulse, administer hypodermics and give baths. We require also a well-ventilated, sunny, bedchamber, in close proximity to a bathroom. This latter is an essential feature. The patient having been assured of the painlessness of the treatment, and his fears allayed, (and should he have ever attempted voluntary relinquishment he needs this assurance) gives into his physician's keeping his hypodermic syringe and surrenders himself to his control. In the first place, all addicted to the use of opium take more than is necessary for their comfort, and a large reduction, usually about one-third, can be made at once without causing any discomfort.

The patient is at the same time informed that he will be allowed a sufficient quantity to keep him comfortable, and if the amount given him does not do this, he may ask for and will receive enough more to satisfy his needs. I wish to enter here my protest against these forcible detentions, the personal searchings, and the insulting surveillance of detectives in the guise of nurses that is customary in these cases. It is a confession of incompetency, an admission of ignorance, and a revival of that barbarism that bound with cords the surgeon's subject to the table. An opium taker is not without honor, and besides do you think any action inexcusable which would give him relief from the physical gehenna to which the usual treatment, or rather lack of treatment, has condemned him? Let us trust him then until he proves unworthy of it, for in the urine we have a watcher that cannot be deceived, and which will truly respond in answer to the proper tests, as to whether opium is being taken or not.

Beginning with the sedatives, we give as an initial dose, twice daily, one drachm of sodium bromide, dissolved in six or eight ounces of cold water, slightly acidulated with the juice of lemons. This method of administration accomplishes two ends: It renders it not unpleasant to the taste and less liable to disturb the stomach. Each dose is increased daily by thirty grains so that on the fifth day, three drachms night and morning are being taken. Slight symptoms of bromism now begin to be manifest, the breath becomes somewhat offensive, an unpleasant odor exhales from the body, and there is a metallic taste in the mouth. There is slight mental hebetude, ideas are evolved slowly and the speech becomes somewhat thickened and the articulation difficult. We welcome these symptoms as indications for a large reduction in morphia and the quantity is rapidly cut down, while we continue to give the bromides at the maximum dose that was being taken at the time when its characteristic effects became prominent. In a few days the narcotic is withdrawn altogether, and now we begin to get the symptoms which follow the discontinuance of opium. But how different is the condition from the one earlier described. The diarrhœa can now be held in check; at no time is it exhaustive. The stomach performs its duties properly, rarely does even nausea supervene, and in place of the agonizing tortures of the former described condition, is substituted a dull listlessness that is neither pain nor pleasure. And now, having accomplished the major part, we may direct our medication to the symptoms with the assurance that the desired effects will follow. Taking the symptoms in detail, restlessness (that invariable sequel to the abandonment of opium, and wanting which the patient is *surely* obtaining, the drug surreptitiously) is best combatted by the hot bath. This should be at a temperature of at least 95° F. After the patient's immersion in it, the temperature is rapidly run up by the addition of hot water, to the point of approaching discomfort. He may remain in the bath from fifteen to twenty minutes, then having been rapidly dried and wrapped in warm blankets, he is put to bed. These baths may be repeated at two, three or four hours interval as may be necessary. But this condition is not of long continuance usually, but for the first day or two after complete withdrawal of the narcotic, and may be surely relieved by the plan I have mentioned.

As I have said, the stomach, under this method of treatment rarely becomes disordered. Should, however, nausea or vomiting set in, they yield readily to small quantities of beef peptonoids, acidulated with a weak solution of phosphoric acid. In the event of disordered stomach, if the diarrhœa is inclined to be excessive, large enemata of hot water will hold it in check, but usually astringents may be given by the mouth, and of these catechu and coto rank first. I prefer the latter given in mxxx doses of the fluid extract, repeated *pro re nata*. Without any treatment, a condition of complete insomnia ensues on the discontinuance of opium and lasts for ten days or more, varying with the amount of the drug taken and length of addiction. Under *any* method of treatment more or less loss of sleep is inevitable, but we have in cannabis indica a reliable hypnotic in these cases, not given in homœopathic dilutions, but boldly in doses of a half drachm of Squibb's fluid extract, repeated if necessary. It is surprising how the nervous system resists narcotic impressions after having been habituated to opium, and small doses are worse than useless, inducing excitement where we want repose. I have specified Squibb's fluid extract, from its being the most reliable preparation of the drug mentioned, and it is best given in capsule. The preceding forms the major part of treatment and will successfully meet the greater disturbances that follow the withdrawal of the opium. The minor indications are, for relief of the fleeting neuralgias, headaches, etc., for the restoration of the loss of general tonicity, and to counteract the depression. For the first mentioned the electric current seems to give the best results, localized galvanization for the neuralgias, and faradization for the less acute neuroses.

General faradization is an excellent tonic *per se*, and may be supplemented by some of the preparations of quinine and nux vomica internally. Coca, while by no means a specific for opium addiction, is a valued aid in treatment. I prefer giving it as cocaine hydrochlorate in ¼ grain doses, as the fluid extract of coca is a nauseous dose and is called for just at the time when the stomach is inclined to be rebellious. The alkaloid will meet all the indications better, and in combination with capsicum will relieve the depression that so often follows the disuse of the narcotic. While the symptoms are being met the bromides are discontinued on a descending scale, as it is better that the system should learn to depend upon itself as soon as possible, and the use of the hypnotics continued no longer than is absolutely necessary, for the same reason. During and following convalescence a tonic treatment is usually required. Iron, quinine, strychnine, cod liver oil, etc., are now in order. A generous diet, nutritious and digestible, is to be ordered or rather permitted, for often the quantity will have to be restricted, else the appetite under the demands of the system for nourishment, might tempt the patient into an indulgence that would overtax his digestion. These points of treatment cover all the important symptoms in this disease. There may be sequelæ that call for separate medication, but these do not differ from those encountered under other circumstances and do not demand physical treatment. There is one condition, however, that deserves more than passing mention. The painful neuralgias which are so frequently the source of the primary addiction, and which are so prone to recur after cure, call for an anodyne without morphia. In these cases I have had satisfactory results from the following:

℞—Antipyrin. .............................. dr. iij.
Ext. Cannabis Indica..........................
Ext. Aconite ............................ aa gr. vss.
Caffein ................................. dr. ss.
Hyoscine Hydrobrom...................... gr 1.

Divide into thirty capsules. Dose one repeated if necessary.

There is a wide-spread opinion, both among medical men and the people generally, that unless recovery is never followed by relapse the treatment has been a failure. This idea is both erroneous and unjust. A patient recovering from malaria, if he be exposed to the same etiological surroundings, will have a recurrence of the disease. Would this prove the primary failure of antiperiodic medication? The same is true of opium addiction. Indeed he risks no increased liability by virtue of an enlarged susceptibility, directly the outcome of his first attack. Hence his only safety lies in utterly abstaining from and repelling the temptation to an occasional indulgence. Levinstein has said and truly, "that one injection will undo a good result successfully kept up for months together."

It should be clearly understood that opium habituation means death, mental, moral and physical, and if ever a poor unfortunate needed help and that quickly it is the opium *habitué*. From whom else but you, as physicians, can he hope for cure, and gentlemen, when under your intelligent treatment, from the haggard, emaciated and sodden wreck, you see him emerging, clear eyed, ruddy, erect, rejoicing again in the fullness of vigorous manhood, you will feel the force of the truth, expressed by the old latin poet: "*In nulla re propius deos homines accedunt quam salutem hominibus dando.*"

